# An integrated analysis of the competing endogenous RNA network associated of prognosis of stage I lung adenocarcinoma

**DOI:** 10.1186/s12885-022-09290-0

**Published:** 2022-02-19

**Authors:** Yuan Xu, Guofu Lin, Yifei Liu, Xianbin Lin, Hai Lin, Zhifeng Guo, Yingxuan Xu, Qinhui Lin, Shaohua Chen, Jiansheng Yang, Yiming Zeng

**Affiliations:** 1 Department of Respiratory Pulmonary and Critical Care Medicine, The Second Affiliated Hospital of Fujian Medical University, Quanzhou, 362000 Fujian province China; 2 Respiratory Medicine Center of Fujian Province, Quanzhou, 362000 Fujian province China; 3 Clinical Center for Molecular Diagnosis and Therapy, The Second Affiliated Hospital of Fujian Medical University, Quanzhou, 362000 Fujian province China; 4 The Second Clinical College, Fujian Medical University, Fuzhou, 350004 Fujian province China; 5 Department of Thoracic Surgery, The Second Affiliated Hospital of Fujian Medical University, Quanzhou, 362000 Fujian province China; 6 Department of Pathology, The Second Affiliated Hospital of Fujian Medical University, Quanzhou, 362000 Fujian province China

**Keywords:** Lung adenocarcinoma, Stage I, lncRNA, ceRNA, FENDRR

## Abstract

**Background:**

Accumulating evidence indicates that long non-coding RNAs (lncRNAs) are involving in the tumorigenesis and metastasis of lung cancer. The aim of the study is to systematically characterize the lncRNA-associated competing endogenous RNA (ceRNA) network and identify key lncRNAs in the development of stage I lung adenocarcinoma (LUAD).

**Methods:**

Totally, 1,955 DEmRNAs, 165 DEmiRNAs and 1,107 DElncRNAs were obtained in 10 paired normal and LUAD tissues. And a total of 8,912 paired lncRNA-miRNA-mRNA network was constructed. Using the Cancer Genome Atlas (TCGA) dataset, the module of ME turquoise was revealed to be most relevant to the progression of LUAD though Weighted Gene Co-expression Network Analysis (WGCNA).

**Results:**

Of the lncRNAs identified, *LINC00639*, *RP4-676L2.1* and *FENDRR* were in ceRNA network established by our RNA-sequencing dataset. Using univariate Cox regression analysis, *FENDRR* was a risk factor of progression free survival (PFS) of stage I LUAD patients (HRs = 1.69, 95%CI 1.07–2.68, *P* < .050). Subsequently, diffe rential expression of *FENDRR* in paired normal and LUAD tissues was detected significant by real-time quantitative (qRT-PCR) (*P* < 0.001).

**Conclusions:**

This study, for the first time, deciphered the regulatory role of *FENDRR*/*miR-6815-5p* axis in the progression of early-stage LUAD, which is needed to be established in vitro and in vivo.

**Supplementary Information:**

The online version contains supplementary material available at 10.1186/s12885-022-09290-0.

## Introduction

Lung cancer remains the prominent contributor of cancer-related mortality, with the worldwide 5-year survival rate of which is around 16.6% [1, 2]. Recently, with the wide application of low-dose computed tomography for early screening and the rapid development of target drugs for genetic mutations, the progress against lung cancer has achieved profound success. As of 2017, the mortality rate of lung cancer dropped from its peak by 51% among males and by 26% among females [2]. Nonetheless, the 5-year survival rate for lung cancer patients is still not well manifested. Additionally, the early-stage patients’ prognosis displays quite disparate from those of advanced-stage, with 5-year survival ranging from 85 to 6% [3], but its recurrence rate is still up to 90% after local resection or radical excision [4].

Currently, non-small cell lung cancer (NSCLC), accounting for approximately 85% of all lung cancer, has been endowed with several therapeutic options, including surgery, chemotherapy, radiation, target therapy and immunotherapy [5]. And surgery is the first choice of curative treatments for the medically operable. Owing to its readily entry into regional lymph nodes and apt to metastasize at an early stage, however, the recurrence rate accounts for approximately 27% to 38% for stage I NSCLC patients [6–8]. Therefore, intensive efforts have been directed to elucidate the molecular mechanism of premalignancy development and progression, and to identify potential molecular signatures for early diagnosis and interception.

Recently, long noncoding RNAs (lncRNAs) have attracted significant attentions in various cancers. LncRNA is a class of transcripts with length of more than 200 nucleotides that possesses limited or no protein-coding capacity, which is transcribed by RNA polymerase II, spliced, 5’capped, and polyadenylated [9]. It has been identified that lncRNAs were involved into diverse cellular, physiological and pathological process via a series of mechanism [10, 11], including serving as critical regulators of tumorigenesis and metastasis [12]. Furthermore, accumulating evidences revealed that lncRNA could disrupt miRNA-mediated degradation of target mRNAs by acting as “miRNA sponges” [13], indicating coding and noncoding RNAs could control one another through their ability to compete for miRNA binding locus, which termed as “ceRNA”. Under this hypothesis, a growing number of evidence revealed that ceRNA axis could contribute to tumorigenesis, progression and metastasis of cancer [14, 15]. For instance, researchers have demonstrated that *LINC00336*, an novel regulator of ferroptosis, could act as a ceRNA to affect tumor genesis and progression and mediate the expression of cystathionine-β-synthase (CBS) by sponging miR6852, which may serve as a potential therapeutic target of lung cancer [16]. Also, a novel lncRNA CCAT1 was reported to directly bind to miR-218 response elements and to promote gefitinib resistance of NSCLC by acting as a ceRNA of miR-218 to regulate HOXA1 expression [17]. However, most of previous studies on lncRNAs acting as ceRNAs were carried out on NSCLC patients with ignoring the heterogeneity of staging, and the clinical significance of tumor progression in early stage remains largely unknown.

Lung adenocarcinoma (LUAD) accounts for the most common subtype of NSCLC, which brings to light the necessity of distinguishing LUAD from other subtypes at an early stage to propose individual treatment. To date, it has been revealed that *EGFR* and *KRAS* mutations, and multiple other oncogenic and tumor suppressor genes were involved in the process of initiation and pathogenesis [18, 19]. Given tumor’s biological behaviors and molecular characteristic displaying heterogeneous at an early stage, some patients could experience a long-term survival, while others could not. To address this, we performed RNA-sequencing for stage I LUAD patients to identify differentially expressed lncRNAs (DElncRNAs) in paired normal and tumor tissues, following by ceRNAs network constructed. On this basis, lncRNA-related ceRNAs identified were validated by database from The Cancer Genome Atlas (TCGA) to establish clinical prognostic model. Furthermore, the hub gene related ceRNAs regulatory network of lncRNAs-miRNAs-mRNAs were established to broaden our knowledge of expression pattern.

## Methods

### Patients and clinical samples

A total of 10 paired LUAD and adjacent normal fresh tissues were obtained from Second Affiliated Hospital of Fujian Medical University between January, 2019 and May, 2019. The specimens were collected from LUAD patients with treatment-naïve who underwent primary surgical treatment. All of the specimens were immediately snap-frozen by liquid nitrogen after surgical resection, and stored at -80℃ until RNA extraction. The clinicopathological diagnosis were confirmed by two pathologists according to the guidelines of the World Health Organization (WHO, version 2015). All participants provided written informed consent, and the bioethical committees at The Second Affiliated Hospital of Fujian Medical University, China, gave written approval for the study (2020–206).

The other group resulted from the LUAD-related RNA-sequencing data in TCGA database, containing the expression data of mRNAs, miRNAs and lncRNAs. A total of 595 paired LUAD tissues RNA-seq data were retrieved, with their clinicopathological features. RNA-sequencing data and corresponding clinical information in LUAD were obtained from TCGA database as following criteria: 1) histologically diagnosed as LUAD; 2) data with complete clinical information; 3) diagnosed as stage I patients. Finally, a total of 260 paired LUAD tissues was included for further analysis. The present study meets the criteria of data usage and publishing of the National Cancer Institute of NIH, and no approval from the ethics committee was required. Progression-Free Survival (PFS) was calculated from the date when patients first received treatment until the date of progression or the last follow-up or death.

### RNA extraction and sequencing

Total RNA of LUAD tissues and corresponding normal tissues was extraction from frozen tissues using RNeasy Mini Kit (Qiagen, Germany) following the manufacturer’s protocol. The RNA concentration was evaluated by the Qubit 4.0 (Thermo Fisher Scientific, Wilmington, DE, USA), and the RNA quality was evaluated by agarose gel eletrophoresis.

Then, ribosomal RNA was removed from total RNA to obtain the maximum residual ncRNA. After fragment of rRNA-depleted RNA, the cDNA library was constructed using the TruSeq RNA sample Prep Kit (Illumina, San Diego, CA, USA). LncRNA/mRNA sequencing libraries were prepared using VAHTS total RNA-seq Library Prep kit for Illumina (Vazyme NR603, China) according to the manufacturer’s protocol. The cDNA fragments with 150-bp paired-end reads were generated for RNA sequencing. Additionally, NEBNext® Multiplex Small RNA Library Prep Set for Illumina® (NEB) was used to prepare the miRNA library for samples. 12 libraries were pooled and sequenced in a single lane of Illumina HiSeq Xten sequencing platform. And Illumina’s TruSeq small RNA library preparation kit was used to prepare the miRNA library with 50-bp paired-end reads generated. After library construction, RNA sequencing for both lncRNA/mRNA and miRNA was performed using Illumina HiSeq Xten platform.

### Identifications of DEmRNAs, DEmiRNA and DElncRNAs

Mirdeep2 (v2.0.0.5) was used for new miRNA prediction, whose expression was calculated and standardized using CPM (counts per million read, CPM). LncRNAs were annotated by the following database, including PLEX (https://sourceforge.net/projects/plek/), CPAT (https://sourceforge.net/projects/rna-cpat/), CNCI (https://github.com/www-bioinfo-org/CNCI) and CPC2 (http://cpc2.cbi.pku.edu.cn/). The intersection was exhibited using Venn diagram. (Figure S 1).

DESeq2Rpackage(https://bioconductor.org/packages/release/bioc/html/DESeq2.html) in Bioconductor project was used to screen the differentially expressed mRNA (DEmRNAs), differentially expressed miRNA (DEmiRNAs) and differentially expressed lncRNA (DElncRNAs) between LUAD and normal tissues. |Log_2_(fold change)| (|log_2_FC|) ≥ 1 and statistical *P* value ≤ 0.05 were set as cut-off criteria. Finally, unsupervised hierarchical clustering was carried out for DE-RNAs, and the expression patterns of which in paired tissues were displayed in form of heatmap using the pheatmap R package (https://cran.r-project.org/web/packages/pheatmap/index.html).

### Analysis of the DElncRNAs Enrichment pathway

Gene ontology (GO, http://www.bioconductor.org/packages/release/bioc/html/topGO.html) analysis was conducted to screen enrichment of target genes to determine the biological functions regulated by lncRNAs, including biological processes (BP), cellular component (CC), and molecular function (MF) annotations. Kyoto Encyclopedia of Genes and Genomes (KEGG, https://www.kegg.jp/kegg/kegg1.html) analysis was performed to annotate the signaling pathways mainly involved for target genes [20, 21]. The GO and pathway analysis with enriched gene count ≥ 2 and *P* value < 0.05 as the threshold for statistical significance.

### Predication of miRNA regulation relationship

The predication of miRNA-gene analysis of DEmiRNA obtained was performed using the online website miRWalk 2.0 (http://zmf.umm.uni-heidelberg.de/pps/zmf/mirwalk2/). Furthermore, miRWalk, miRanda, miRDB, miRMap and TargetScan databases were used to predict the possible DEmiRNA-DEmRNA regulatory relationships. Using the StarBase (http://starbase.sysu.edu.cn/) database, the miRNA-lncRNA regulatory relationships by DEmiRNA were predict. Then the DEmRNA-DEmiRNA and DEmiRNA-DElncRNA regulatory regulation relationship were constructed based on shared miRNAs with which both lncRNAs and mRNAs interact, and were illustrated by Cytoscape software.

### Construction of lncRNA-miRNA-mRNA ceRNA network

Under the hypothesis of miRNA sponge, the positive correlation expression of lncRNAs-mRNAs was focused on, therefore, and the positive correlation coexpression relationship between mRNAs and lncRNAs simultaneously regulated by miRNAs was obtained. ceRNA network were constructed based on shared miRNAs with which mRNAs and lncRNAs interact. For further analysis, we used a hypergeometric cumulative distribution function test to determine potential ceRNA pairs [22], with correlation coefficient > 0.5 and *P* value < 0.05 as a ceRNA triplet with statistical significance.

### Weighted gene correlation network analysis (WGCNA)

As described previously [23, 24], gene co-expressed network analysis was performed on LUAD and adjacent normal tissues using R WGCNA package. The expression matrix was restricted to expressed lncRNAs, following by a Pearson correlation matrix and a weighted adjacency matrix generated. The module eigengene (ME) was calculated for each module, the values of which clinical traits associated with was calculated by Pearson’s correlation. In the present study, we set the optimal soft-thresholding power at 4 (scale-free *R*^*2*^ = 0.85), cut height at 0.25, and minimal modules sized to 30, to identify key modules. topological overlap matrix (TOM) was constructed and transformed. Then, the lncRNAs were grouped into co-expression modules using tree pruning of gene hierarchical clustering dendrograms by cutreeDynamic method, with correlated modules merged. The module significantly correlated with sample traits were used for hub genes selection.

### LUAD-specific prognostic lncRNA signatures identification

Progression-free survival (PFS) time was calculated from the date when patients first received chemotherapy until the date of progression or the last follow-up or death. Using TCGA dataset, the associations between DElncRNAs in ceRNA network and PFS in stage I LUAD patients were evaluated using univariate Cox regression analysis. The samples were divided into high expression and low expression groups based on the median FPKM of lncRNAs. Kaplan–Meier (KM) survival analysis and Log-rank (LR) test were performed, and LR *P* value, hazard ratio (HR) with 95% confidence interval (CI) were computed. Survival and survminer R package were used for Cox regression analysis, and the statistical *P* < 0.05 was considered as significant.

### RNA extraction and quantitative real-time RT-PCR assay

Total RNA was extracted using (Trizol®; Sigma) from 26 paired LUAD tissues, according to the manufacturer’s recommendations. After purification, the RNA was transcribed into cDNA using Reverse Transcription Kit (Takara, Tokyo, Japan). RT-PCR analysis was performed using the SYBR Green (Takara). The primers used for RT-PCR were: FENDRR primers were AGTCACAGCACCAGAAAGCCAAC (sense) and TGATGTTCTCCTTCTT GCCTCAGC (anti-sense); LINC00639 primers were GTGAGTGTTCAGACATGCCAGGAG (sense) and AGCCGAGTGGATTCAGCGAGAG (anti-sense); RP4-676L2.1 primers were TGTTTGAAGCCGTGAGACTGAGTG (sense) and CTCCTTTGCTGGCTC TTCCTCATC (anti-sense); GAPDH primers were CTCCTGCACCACCAACTGCTTAG (sense) and GACGCCTGCTTCACCACCTTC (anti-sense); miR-6815-5p primers were AGGTGGCGCCGGAGGA (sense) and AGTGCAGGGTCCGAGGTATT (anti-sense); U6 primers were CTCGCTTCGGCAGCACA (sense) and AACGCTTCACGAATTTGCGT (anti-sense). The cycle conditions were as follows: 95 °C 10 min, followed by 40 cycles of 95 °C for 15 s, 65 °C for 30 s, and 72 °C for 30 s. Relative quantification was calculated using the 2^−ΔΔCT^ method.

## Results

### Identification of DEmRNAs, DEmiRNAs and DElncRNAs

**Fig. 1:**
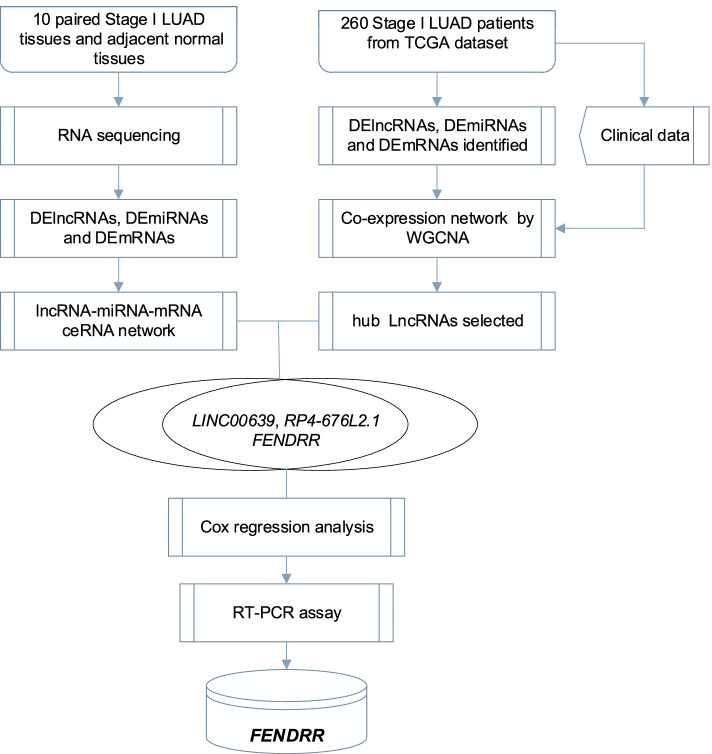
The flowchart of the present study. DElncRNAs: differentially expressed lncRNA; DEmiRNAs: differentially expressed miRNA; DEmRNAs: differentially expressed mRNA; WGCNA: Weighted gene correlation network analysis

**Fig. 2:**
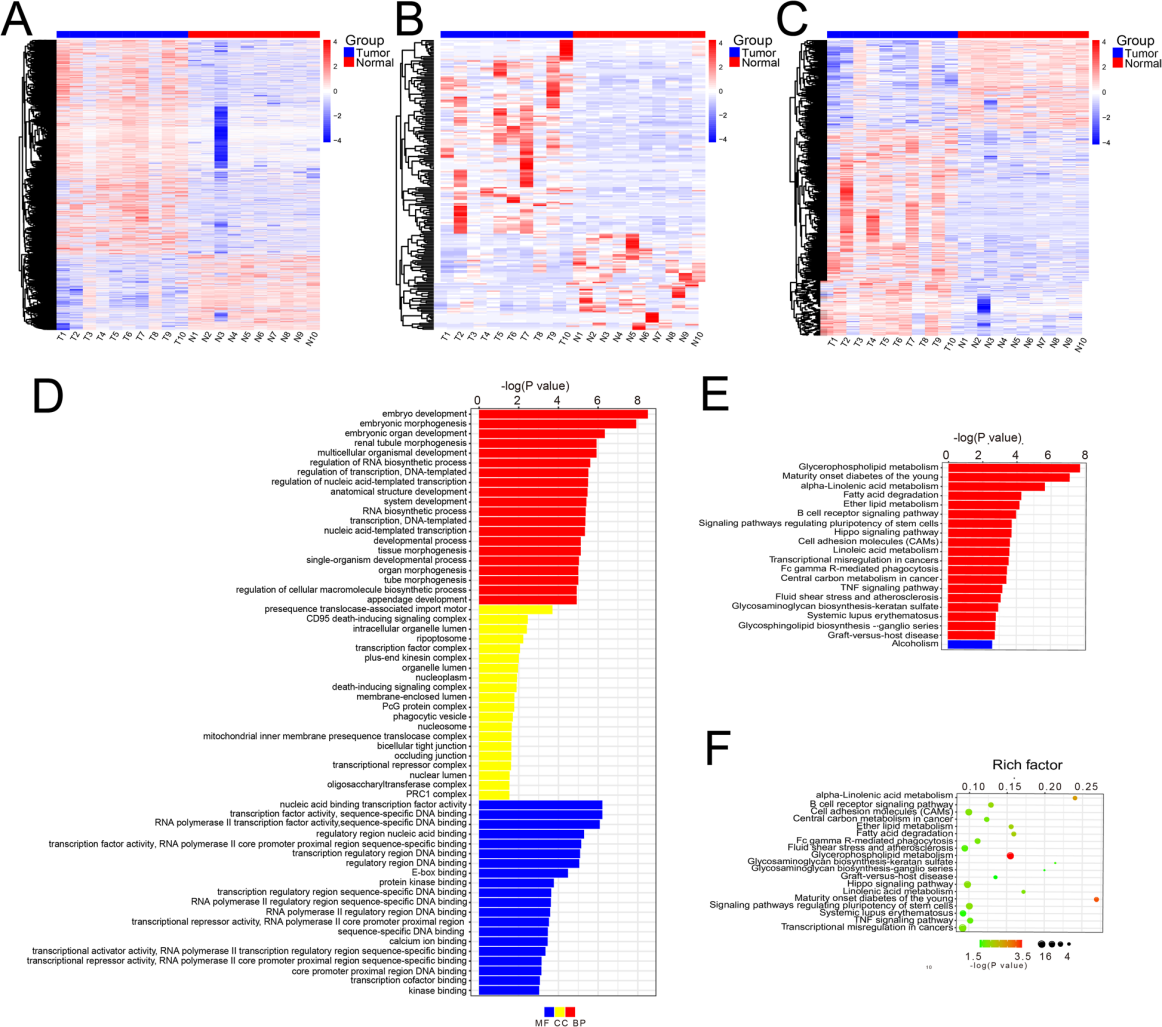
Analysis of expression profile and function enrichment. **A-C** The heatmaps of DEmRNAs, DEmiRNAs and DElncRNAs in the expression profile in 10 paired LUAD and adjacent normal tissue. **D** Top 20 GO terms enriched in DElncRNAs. **E–F** Top 20 pathways enriched in DElncRNAs using Kyoto Encyclopedia of Genes and Genomes (KEGG) analysis and displayed in bubble chart (included predominant metabolic pathway—map 00,564). BP: biological processes; CC: cellular component; MF: molecular function

The schematic diagram was constructed to display the methods of the present study (Fig. 1). Initially, to explore the role of lncRNAs in stage I LUAD, we performed RNA-seq analysis of total RNA in 10 paired tumor and adjacent normal tissues. According to the criteria of |log_2_FC|≥ 1 and *P* value ≤ 0.05 cut-off, a total of 1,955 DEmRNAs (1,372 up- and 583 down-regulated), 165 DEmiRNAs (111 up- and 54 down-regulated) and 1,107 DElncRNAs (775 up- and 332 down-regulated) were detected from dataset. The heatmaps of DEmRNAs, DEmiRNAs and DElncRNAs were presented in Fig. 2A-C, and the top 20 of which were displayed in Table S1, 2, 3, respectively.

To explore the significance of DElncRNAs that might involve in LUAD, we performed functional enrichment analysis of target mRNAs. It implied that the most significant BP, CC and MF by DElncRNAs enriched were embryo development (GO:0,009,790, *P* = 3.10E-09), presequence translocase-associated import motor (GO:0,001,405, *P* = 0.0002) and nucleic acid binding transcription factor activity (GO:0,001,071, *P* = 6.10E-07), respectively. Figure 2D displayed the top 20 GO terms enriched by DElncRNAs. Additionally, pathway enrichment analysis was performed to reveal potential biological function of DElncRNAs. It showed that the top 20 pathways were significantly enriched using Kyoto Encyclopedia of Genes and Genomes (KEGG) analysis, with glycerophospholipid metabolism pathway enriching the most significant genes in the network (Fig. 2E and F).

### ceRNA network construction

**Fig. 3:**
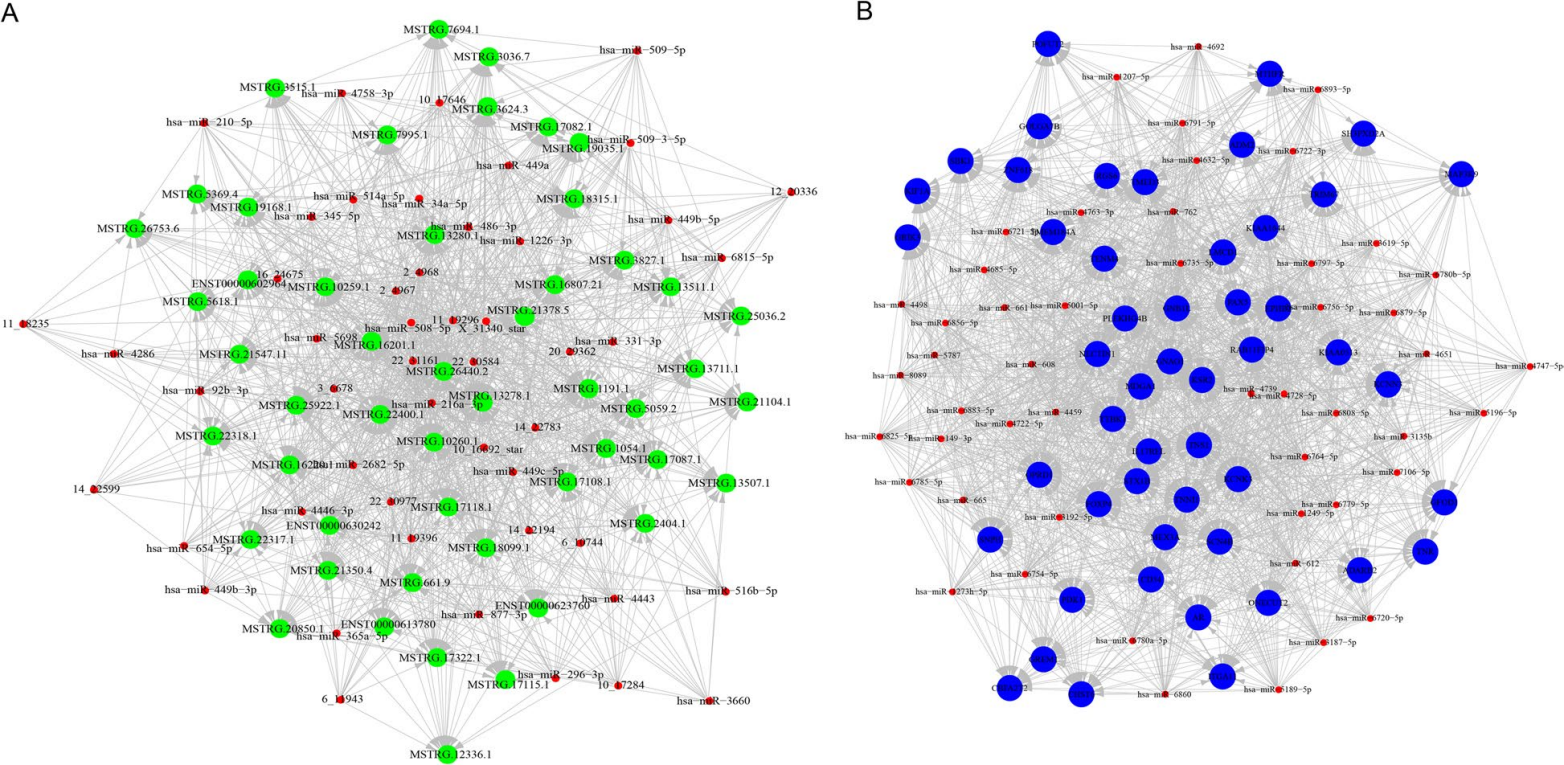
Construction of ceRNA network. **A-B** The regulation relationship between miRNA and mRNA predicted by miRanda, and the relationship between miRNA and lncRNA predicted by miRanda, with the criteria of total score ≥ 150 and total energy ≤ -20. The green node represents lncRNAs; the red node represents miRNAs; the blue node represents mRNAs

With the criteria of total score ≥ 150 and total energy ≤ -20, we used miRanda to predict the regulation relationship between miRNA and mRNA, as well as the relationship between miRNA and lncRNA (Fig. 3A and B). According to the regulation of DElncRNA-DEmiRNA and DEmRNA-DEmiRNA, lncRNAs and mRNAs regulated by the same miRNA were screened. Using the parameter of Pearson correlation (PPC) > 0.5 and *P* value < 0.05 as a threshold, a total of 8,912 interaction relationships of lncRNA-miRNA-mRNA with statistical significance were predicted in 10 paired LUAD tissues, and the top 20 ceRNA network were listed in Table S4.

### Co-expression module identification and hub gene selection

**Fig. 4:**
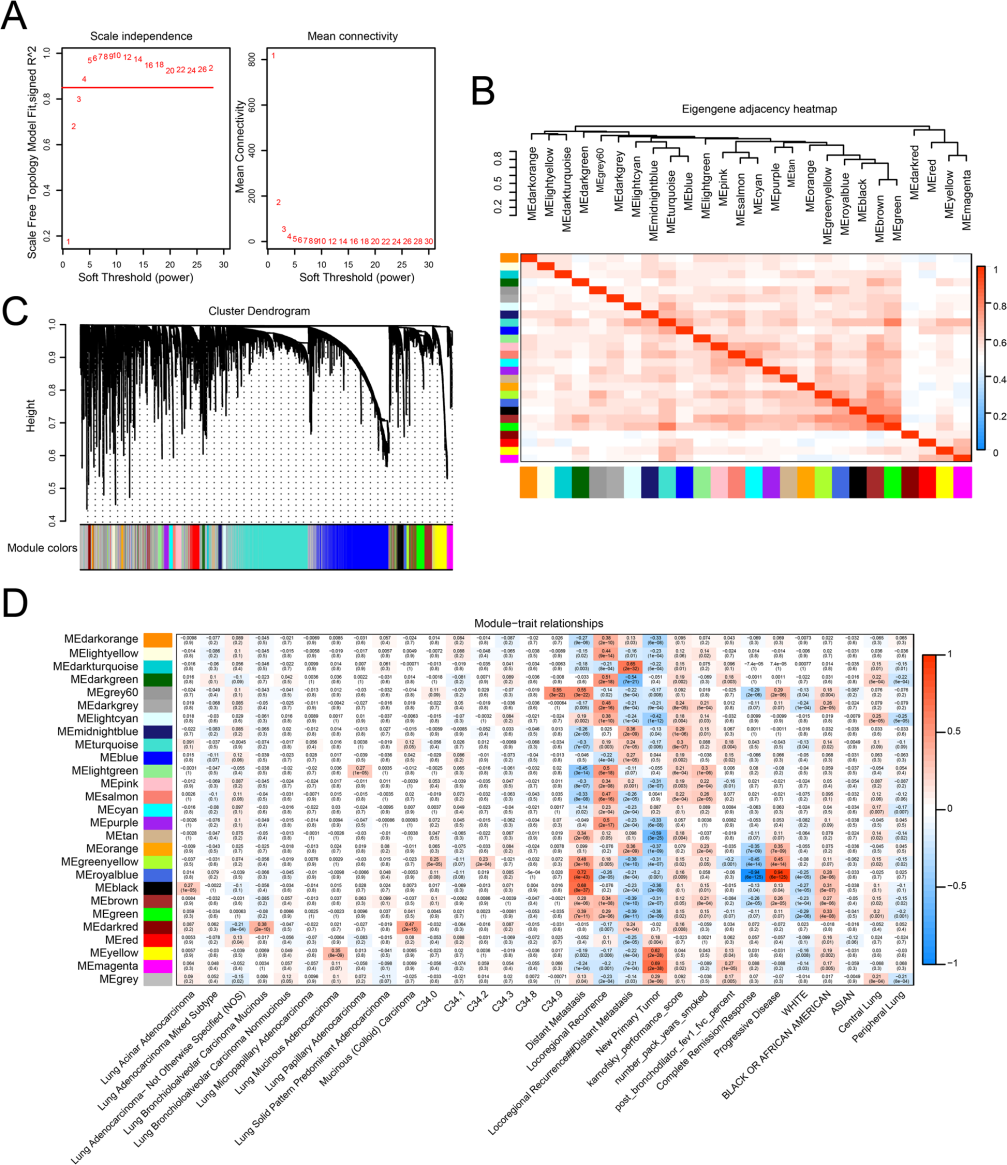
WGCNA and identification of significant modules. **A** The soft-thresholding power in WGCNA. **B** The eigengene of each colored module were calculated to establish an adjacent matrix. **C** Cluster dendrogram obtained from lncRNAs data of stage I LUAD in TCGA dataset with average hierarchical linkage clustering. The color row underneath the dendrogram represents the module assigned by Dynamic Tree Cut. **D** Module-trait relationship heatmap. The row represents the modules, while the column represents the trait. The values in the box represents the correlation and *P* values

To further explore the potential function of lncRNAs in the progression of LUAD, the transcriptomic data of 260 stage I LUAD patients and their corresponding clinical data in TCGA database were extracted to construct WGCNA network. In this study, we chose β = 4 (*R*^*2*^ = 0.85) as a soft threshold to construct a scale-free topology network (Fig. 4A). The TOM including all genes was depicted by topological overlapping heat map. Totally, 43 gene co-expression modules were identified by linkage hierarchical clustering according to TOM-based dissimilarity measure (1-TOM). Finally, we identified 7 modules relevant to clinical traits, as eigengene adjacency heatmap descripted (Fig. 4B). Of these, the turquoise module was most highly correlated with DElncRNAs (Fig. 4B and C). Interestingly, we found that the correlation coefficient between royalblue module and progressive disease reached to 0.94, indicating that the royalblue module is a gene set specifically associated with progression of disease. The royalblue module is also the most relevant module to distant metastasis (cor = 0.72), indicating the correlation of lncRNAs with prognosis (Fig. 4D). By setting the module membership (MM) to > 0.8 and the gene significance (GS) to > 0.4 as threshold, we selected a total of 81 hub lncRNAs from the modules (Table S 5), most of which were involved in the recurrence or metastasis of disease. The results indicated that the DElncRNAs identified were involved in the prognosis of stage I LUAD patients.

**Fig. 5:**
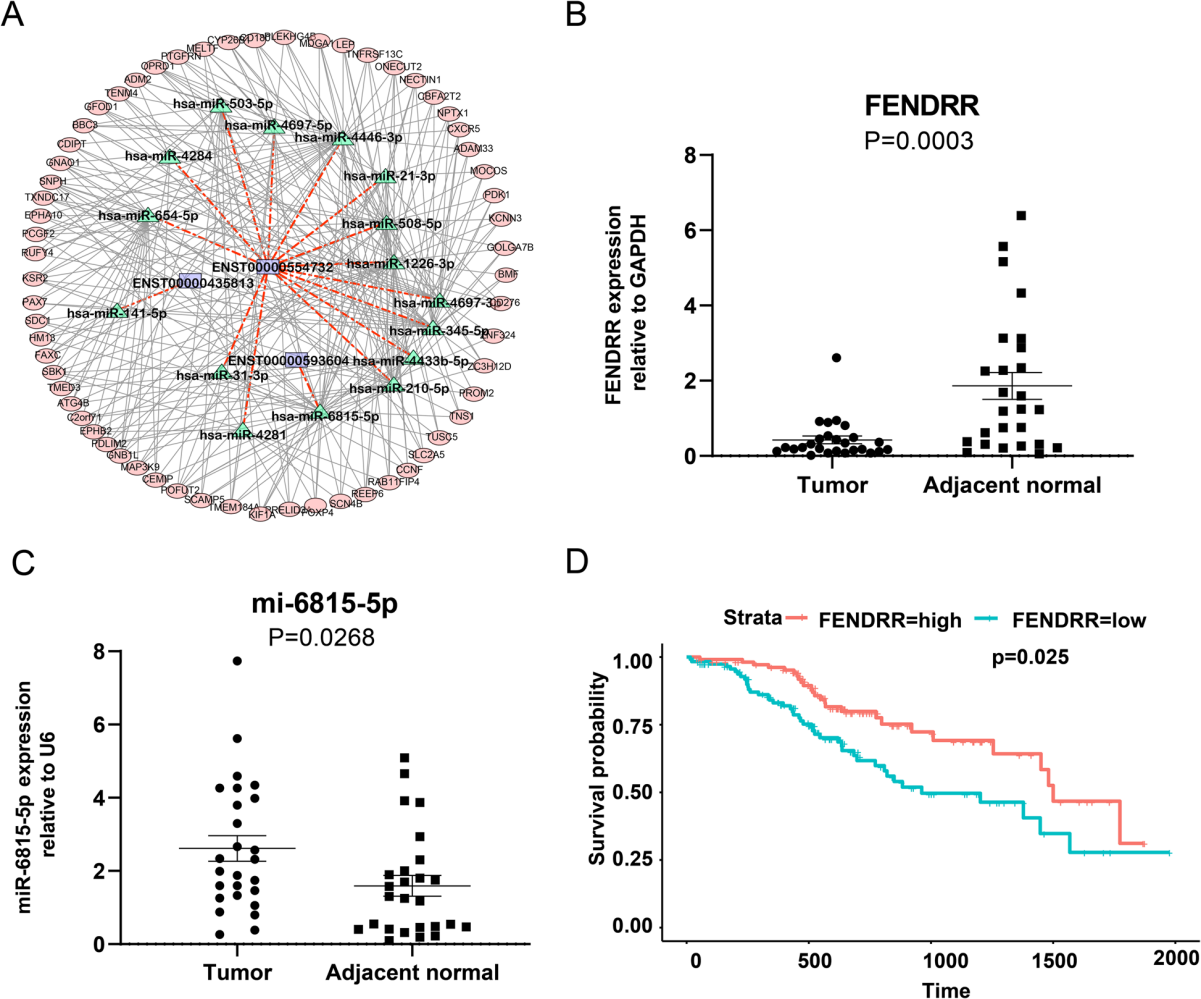
The relationship and expression between lncRNAs and miRNAs. **A** The ceRNA network constructed by *FENDRR*, *LINC00639* and *RP4-676L2.1*. **B-C** The *FENDRR* and miR-6815-5p expression levels of stage I LUAD tissues and paired normal tissues were tested by RT-PCR (*n* = 26). **D** Prognostic significance of *FENDRR* expression on PFS for stage I LUAD patients by the median value as cutoff

Subsequently, we found that 19 genes were associated with distant metastasis, 5 genes associated with locoregional recurrence and 11 genes associated with distant metastasis, and another 65 genes associated with new primary tumor. Of the hub genes identified, *FENDRR*, *LINC00639* and *RP4-676L2.1* were also predicted as ceRNAs in stage I LUAD constructed by our database. Furthermore, we constructed ceRNA network using the three lncRNAs by Cytoscape (Fig. 5A). Finally, ceRNA network containing 174 lncRNA-miRNA-mRNA regulatory relation for *FENDRR*, 937 lncRNA-miRNA-mRNA regulatory relation for *LINC00639*, and 54 regulatory relation for *RP4-676L2.1*. It showed that and *LINC00639* were hub nodes that could target more miRNAs and mRNAs in the network. These findings indicated that DElncRNAs regulated mRNA expression via interaction with miRNAs.

Interestingly, one of subnetworks showed that lncRNA *FENDRR* only act as a sponge for hsa-miR-6815-5p to regulated mRNAs, including TNS1, PDLIM2, PPFIBP1, SCMH1, PLXDC1 and so on, which mainly involved in adherens junction, chromatin silence, blood vessel development (Figure S2A). Thus, we further explore the relationship between *FENDRR and* hsa-miR-6815-5p. In our analysis of RNA sequencing, the results showed that *FENDRR* and *LINC00639* were significantly downregulated, while RP4-676L2.1 and hsa-miR-6815-5p was marly upregulated in stage I LUAD patients (*P* value < 0.05, Figure S 2B). Further, RT-PCR indicated that expression of *FENDRR* was significantly decreased and expression of hsa-miR-6815-5p was markedly increased in LUAD tissues compared with paired normal tissues (*P* value < 0.001) (Fig. 5B and C), while the expression of *RP4-676L2.1* and *LINC00639* were not significant (Figure S2C). Thus, the results revealed that down-regulation of FENDRR maybe involved in tumorigenesis via upregulating of hsa-miR-6815-5p.

To further explore the *FENDRR* identified, we evaluated its effects on recurrence by univariate Cox regression analysis in TCGA dataset. It showed that *FENDRR* was significantly associated with PFS (HRs = 1.69, 95%CI 1.07–2.68, *P* < 0.05), indicating that *FENDRR* could be regarded as a prognostic factor of tumor recurrence. Using median FPKM of *FENDRR* as cutoff value, we divided the patients into high-expression group and low-expression group. KM survival curve for the two groups indicated that PFS in the high *FENDRR* expression group displayed significantly longer than that in the low *FENDRR* expression group (Fig. 5D), while PFS for *RP4-676L2.1* and *LINC00639 between* two groups were not significantly different (Figure S2D).

## Discussion

Although it has achieved great improvement on clinical management for LUAD aided by the discovery of genetic mutations, there is still main obstacles on improving prognosis of patients resulting from its apt to migration and invasion. Therefore, the promise of utilization of lncRNAs in predicating clinical prognosis has attracted much attention in translational research. In this study, we identify three lncRNAs, *FENDRR*, *LINC00639* and *RP4-676L2.1*, which might be related with prognosis of stage I LUAD patients. However, only *FENDRR* is predicted to be associated with PFS of patients, and validated to be down-regulated in LUAD tissues by RT-PCR assay. The primary results uncover the potential role of *FENDRR* in the progression of early-stage LUAD. Importantly, the study gives a novel hint of the mechanism by which *FENDRR* might involve in the progression of disease.

Previous studies have revealed that lncRNAs could exert their biological function via interaction with DNA, RNA and protein depending on their location within cells [25]. Although there is still a lot to learn about lncRNA, accumulating studies demonstrated its function on relieving target mRNAs degradation mediated by miRNA, playing a role as ceRNAs, which could act as vital regulators in various physiological and pathological process of tumor [26, 27]. To date, despite the plethora of reports, several lncRNAs have been identified to be involved in recurrence and metastasis of lung cancer [28–30]. In this study, we explored the most likely significant lncRNAs profile alternations of stage I LUAD, and revealed that *FENDRR*, *LINC00639* and *RP4-676L2.1* were predicted to be associated with progression of disease. However, only *FENDRR* were validated as predictive lncRNA of PFS by cox regression analysis, indicating its underlying function responsible for progression of LUAD.

LncRNA fetal-lethal non-coding developmental regulatory RNA (FENDRR), also named as FOXF1-AS1, is an intergenic lncRNA with consisting of seven exons. It is located at 3q13.31, 1,354 bp upstream of transcriptional start site, which is transcribed from a bidirectional promoter shared with the protein coding gene *Foxf1* and *Pitx2*. Previous studies revealed that *FENDRR* could regulate cell migration, invasion, and lymphatic metastasis, demonstrating its inhibitory regulation in tumor progression [31]. It was reported that *FENDRR* was highly expressed in the lung, while was lowly expressed in the liver, colon and brain tissues, and the level of which was associated with prognosis of patients [32]. In breast cancer, the low level of *FENDRR* was associated with poor prognosis of patients, including a shorter survival and a shorter PFS [33]. Also, *FENDRR* was found to be related with survival of gastric cancer patients, the expression of which could be suppressed in gastric cancer associated fibroblasts by hypermethylation [34]. In agreement with previous studies [31], the level of *FENDRR* expression in tumor was also shown to be suppressed compared with paired normal tissue.

The mechanism of anti-malignant effects for *FENDRR* might involve in inhibiting cell migration, invasion and mediated stem-like properties by regulating epithelial mesenchymal transition (EMT) [35–37]. Previous studies reported that *FENDRR* could anchor PRC2 and/or TrxG/MLL complexes at its target promoters, increasing PRC2 occupancy and H2K27 trimethylation, which lead to the attenuation of target gene expression [38]. In gastric cancer, *FENDRR* was found to increase cell migration and invasion via up-regulation of FN1, MMP2 and MMP9 [34]. In vitro, *FENDRR* was revealed to decrease the IC50 for cisplatin in A549/DDP cells, and depressed chemotherapy resistance to cisplatin in NSCLC [37]. Hitherto, the contribution of *FENDRR* on progression of LUAD has not been well clarified. In our dataset, *FENDRR* could targeted miR-6815-5p in ceRNA network constructed, suggesting that it might exert function on regulation expression of target mRNAs by ‘sponging’ *miR-6815-5p*. To date, only one study was found that miR-6815-5p was significantly upregulated in exosomes of HPV16-infected cervical-vaginal fluid (CVF) based on microarray analysis, while its biological functions was not reported and unclear [39]. Hence, further studies on miR-6815-5p are warranted. To the best our knowledge, this is the first study where predicts the regulation of *miR-6815-5p* on targeted mRNAs in LUAD. Apart from the predication of lncRNAs on PFS, the main breakthrough of our study is centred around the regulation of *miR-6815-5p* on *FENDRR*, which needs to explore its molecular mechanisms to increase further confidence to this result.

Regarding *RP4-676L2.1* and *LINC00639*, we speculated that they could be related with recurrence of disease by WGCNA using TCGA data set, suggesting their prognostic value in stage I LUAD. However, they were not significantly correlated with PFS of stage I LUAD patients by cox regression analysis. Surprisingly, RP4-676L2.1 and LINC00639 were also not successfully validated to be consistent difference of expression between tumor and normal lung tissue by qPCR. We speculated that it may lie with the bioinformatic analysis. Still, further studies are needed to validate using larger samples.

The discovery of lncRNAs contributes significantly to clinical prognosis in stage I LUAD patients. Its strength lies in not only testing using the sequencing dataset, but also validating using TCGA dataset by bioinformatics methods. Still, some limitations must be noted. Firstly, we speculated that FENDRR/miR-6815-5p axis may play an important role in the biological behavior of LUAD based on bioinformatics analysis and RT-PCR, but the specific mechanism still needs to be further verified such as the functional experiments of LUAD cells and RNA-RNA interaction verification experiments. Secondly, due to the low number of samples performed RNA-seq, the WGCNA was constructed using TCGA dataset, which might induce racial bias. This is not surprising, a substantial number of studies on lncRNAs have been validated using TCGA dataset. Thirdly, the lncRNAs identified from RNA-seq were inconsistent with the qPCR results, which may result from incorrect annotation in the bioinformatic analysis.

## Conclusions

Taken together, this study contributed significantly to the wider knowledge of lncRNAs and ceRNAs involvement of progression of stage I LUAD. Out of the three lncRNAs validated, *FENDRR* is validated to involve in the recurrence of disease, which confirm previous findings. Based on the *FENDRR-*related ceRNA network constructed, it was revealed the regulation of *miR-6815-5p* on *FENDRR* for the first time, therefore open to further research to explore molecular mechanism. However, studies are still needed to establish the role of *FENDRR*/*miR-6815-5p* axis in the progression of early-stage LUAD.

## Supplementary Information

**Additional file 1: Table S1.** Top 20 DEmRNAs identified between tumor and adjacent normal tissues in stage I LUAD patients. **Table S2.** Top 20 DEmiRNAs identified between tumor and adjacent normal tissues in stage I LUAD patients. **Table S3.** Top 20 DElncRNAs identified between tumor and adjacent normal tissues in stage I LUAD patients. **Table S4.** Top 20 ceRNA network constructed in 10 stage I LUAD patients. **Table S5.** Hub lncRNAs predicted by WGCNA using TCGA dataset.

**Additional file 2.**

**Additional file 3.**

**Additional file 4.**

## Data Availability

The datasets generated during the current study are not publicly available due to concerns regarding patient confidentiality and proprietary information but are available upon reasonable request from the corresponding author. we provided a reviewer link of unpublished BioProject. Use the following URL: https://dataview.ncbi.nlm.nih.gov/object/PRJNA732584?reviewer=jo5ph00rjrkr7u19thhft8eho.

## Abbreviations

lncRNAs: Long non-coding RNAs
NSCLC: Non-small cell lung cancer
LUAD: Lung adenocarcinoma
ceRNA: Competing endogenous RNA
PFS: Progression free survival
CBS: Cystathionine-β-synthase
RIG: Retinoid-inducible protein
TCGA: The Cancer Genome Atlas
DElncRNAs: Identify differentially expressed lncRNAs
KEGG: Kyoto Encyclopedia of Genes and Genomes
BP: Biological processes
CC: Cellular component
MF: Molecular function
WGCNA: Weighted gene correlation network analysis
KM: Kaplan–Meier
HR: Hazard ratio
FENDRR: Fetal-lethal non-coding developmental regulatory RNA
