# Supplementary material for: An integrated analysis of the competing endogenous RNA network associated of prognosis of stage I lung adenocarcinoma
Source: BMC Cancer. 2022 Feb 19;22:188. doi: 10.1186/s12885-022-09290-0 (PMC8857797; doi:10.1186/s12885-022-09290-0)
Supplement: Supplementary file 1 — Additional file 1: Table S1. Top 20 DEmRNAs identified between tumor and adjacent normal tissues in stage I LUAD patients. Table S2. Top 20 DEmiRNAs identified between tumor and adjacent normal tissues in stage I LUAD patients. Table S3. Top 20 DElncRNAs identified between tumor and adjacent normal tissues in stage I LUAD patients. Table S4. Top 20 ceRNA network constructed in 10 stage I LUAD patients. Table S5. Hub lncRNAs predicted by WGCNA using TCGA dataset. [file 12885_2022_9290_MOESM1_ESM.docx]

***Title Page***

**An integrated analysis of the competing endogenous RNA network associated of prognosis of stage I lung adenocarcinoma**

Yuan Xu^1,2*^, Guofu Lin^1,2,4 *^, Yifei Liu^3*^, Xianbin Lin^5*^, Hai Lin^1,2,4^, Zhifeng Guo^1,2,4^, Yingxuan Xu^1,2,4^, Qinhui Lin^1,2^, Shaohua Chen^6^, Jiansheng Yang^5^, Yiming Zeng^1,2^

^1.^Department of Respiratory Pulmonary and Critical Care Medicine, The Second Affiliated Hospital of Fujian Medical University, Quanzhou, Fujian province, 362000, China

^2.^Respiratory Medicine Center of Fujian Province, Quanzhou, Fujian province, 362000, China

^3.^Clinical Center for Molecular Diagnosis and Therapy, The Second Affiliated Hospital of Fujian Medical University, Quanzhou, Fujian province, 362000, China

^4.^The Second Clinical College, Fujian Medical University, Fuzhou, Fujian province, 350004, China

^5.^[Department of thoracic surgery](javascript:;), The Second Affiliated Hospital of Fujian Medical University, Quanzhou, Fujian province, 362000, China

^6.^Department of Pathology, The Second Affiliated Hospital of Fujian Medical University, Quanzhou, Fujian province, 362000, China

^*^ Those authors contributed equally to this work.

**Correspondence to**: Prof. Yiming Zeng, The Second Affiliated Hospital of Fujian Medical University, Department of Respiratory Pulmonary and Critical Care Medicine, Quanzhou, Fujian, China; Fujian Respiratory Medicine Center, Quanzhou, Fujian 350004, China. Tel: 86-595-22791001 [**zeng_yiming @fjmu.edu.cn**](mailto:zeng_yiming@fjmu.edu.cn)

| **Table S1**. Top 20 DEmRNAs identified between tumor and adjacent normal tissues in stage I LUAD patients | | | | | | | | | | |  |
| --- | --- | --- | --- | --- | --- | --- | --- | --- | --- | --- | --- |
| **Transcript_ID** | **Gene Symbol** | | | **Normal** | | **Tumor** | | **Log2 Fold Change** | | ***P* value** | |
| ENST00000366518 | | *KIF26B* | 0 | | 8.654592175 | | 8.654592175 | | 3.18E-15 | |  |
| ENST00000360804 | | *SPP1* | 1.320555931 | | 8.835276043 | | 7.514720112 | | 0.020145025 | |  |
| ENST00000261187 | | *SLC16A7* | 0.82421325 | | 8.2987751 | | 7.474561849 | | 2.76E-17 | |  |
| ENST00000512690 | | *RPS3A* | 0 | | 7.292023165 | | 7.292023165 | | 0.037093927 | |  |
| ENST00000222271 | | *COMP* | 0.350903824 | | 7.468774449 | | 7.117870625 | | 6.18E-12 | |  |
| ENST00000616821 | | *HIP1* | 0 | | 6.740645236 | | 6.740645236 | | 6.60E-44 | |  |
| ENST00000304749 | | *CST1* | 0.218430196 | | 6.734197789 | | 6.515767593 | | 1.48E-17 | |  |
| ENST00000393664 | | *ETV4* | 0.65789736 | | 7.150091332 | | 6.492193971 | | 0.002070202 | |  |
| ENST00000403444 | | *CEACAM1* | 2.265297755 | | 8.593524293 | | 6.328226538 | | 0.000448709 | |  |
| ENST00000614399 | | *MUC3A* | 0.190539439 | | 6.414480653 | | 6.223941214 | | 1.16E-23 | |  |
| ENST00000589620 | | *FUT3* | 0 | | 6.223727251 | | 6.223727251 | | 7.03E-25 | |  |
| ENST00000296695 | | *SPINK1* | 1.005817309 | | 7.075748308 | | 6.069930998 | | 5.32E-13 | |  |
| ENST00000406696 | | *HS6ST2* | 0 | | 6.004575254 | | 6.004575254 | | 2.49E-08 | |  |
| ENST00000355153 | | *ITGB2* | 0 | | 5.865750235 | | 5.865750235 | | 3.75E-11 | |  |
| ENST00000398292 | | *GGT5* | 2.115405239 | | 7.698799642 | | 5.583394403 | | 0.018506869 | |  |
| ENST00000351270 | | *HABP2* | 2.698763657 | | 8.256956396 | | 5.558192739 | | 2.44E-13 | |  |
| ENST00000542321 | | *RGMA* | 1.795660395 | | 7.32502676 | | 5.529366365 | | 0.005210264 | |  |
| ENST00000357639 | | *ENPP3* | 1.988295986 | | 7.497581251 | | 5.509285265 | | 0.009093016 | |  |
| ENST00000523206 | | *INTS8* | 5.576337519 | | 0 | | -5.576337519 | | 2.03E-08 | |  |
| ENST00000307765 | | *RXFP1* | 7.586207045 | | 1.173590046 | | -6.412616999 | | 0.001788919 | |  |

| **Table S2**. Top 20 DEmiRNAs identified between tumor and adjacent normal tissues in stage I LUAD patients | | | | |
| --- | --- | --- | --- | --- |
| **miRNA** | **Normal** | **Tumor** | **Log2 FoldChange** | ***P* value** |
| *hsa-miR-1269b* | 0.1 | 5.7 | 5.832890014 | 2.28E-15 |
| *hsa-miR-196a-5p* | 1.6 | 26.7 | 4.060695932 | 1.65E-18 |
| *hsa-miR-6510-3p* | 0.2 | 3 | 3.906890596 | 3.92E-09 |
| *hsa-miR-509-3p* | 0.2 | 3 | 3.906890596 | 2.02E-08 |
| *hsa-miR-508-5p* | 0.1 | 1.3 | 3.700439718 | 0.000282765 |
| *hsa-miR-183-3p* | 0.1 | 1.2 | 3.584962501 | 2.34E-05 |
| *1_2920* | 0.2 | 2.3 | 3.523561956 | 5.47E-07 |
| *hsa-miR-509-3-5p* | 2.7 | 30.7 | 3.507207343 | 3.29E-13 |
| *hsa-miR-514a-5p* | 0.2 | 2.1 | 3.392317423 | 1.40E-05 |
| *hsa-miR-31-3p* | 0.2 | 2 | 3.321928095 | 5.29E-05 |
| *10_16692* | 0.4 | 3.7 | 3.209453366 | 1.23E-06 |
| *hsa-miR-4697-3p* | 0.2 | 1.8 | 3.169925001 | 1.42E-05 |
| *hsa-miR-509-5p* | 0.1 | 0.9 | 3.169925001 | 0.000880139 |
| *hsa-miR-891a-5p* | 0.1 | 0.8 | 3 | 0.000504201 |
| *14_22599* | 10.2 | 1.3 | -2.971985624 | 4.55E-08 |
| *hsa-miR-518a-3p* | 0.8 | 0.1 | -3 | 0.005572009 |
| *18_26720* | 0.9 | 0.1 | -3.169925001 | 0.000216652 |
| *11_19396* | 0.9 | 0.1 | -3.169925001 | 0.000787025 |
| *22_31161* | 1.3 | 0.1 | -3.700439718 | 6.62E-06 |
| *hsa-miR-1973* | 2.2 | 0.1 | -4.459431619 | 3.70E-06 |

| **Table S3**. Top 20 DElncRNAs identified between tumor and adjacent normal tissues in stage I LUAD patients | | | | |
| --- | --- | --- | --- | --- |
| **Transcript_ID** | **Normal** | **Tumor** | **Log2 FoldChange** | ***P* value** |
| *MSTRG.21547.11* | 1.461945401 | 7.335074859 | 5.873129458 | 1.61E-10 |
| *MSTRG.21547.12* | 0.670834747 | 6.401268947 | 5.7304342 | 3.83E-08 |
| *MSTRG.21102.2* | 0 | 5.66306219 | 5.66306219 | 7.61E-08 |
| *MSTRG.19902.1* | 1.633206958 | 6.766937436 | 5.133730479 | 3.29E-08 |
| *MSTRG.22930.1* | 0.212638956 | 5.203134195 | 4.990495239 | 1.01E-11 |
| *MSTRG.22930.2* | 1.357712976 | 6.305162386 | 4.94744941 | 3.75E-06 |
| *MSTRG.17733.2* | 1.764799454 | 6.563233146 | 4.798433693 | 8.50E-05 |
| *MSTRG.14655.4* | 1.698819362 | 6.470141899 | 4.771322538 | 0.000224038 |
| *MSTRG.14626.8* | 0.348765576 | 5.106770045 | 4.758004468 | 3.88E-06 |
| *MSTRG.23939.1* | 0.793969427 | 5.392062653 | 4.598093226 | 2.28E-12 |
| *MSTRG.14654.4* | 2.032638869 | 6.617754019 | 4.58511515 | 2.86E-09 |
| *MSTRG.14628.2* | 4.512240474 | 8.994073946 | 4.481833472 | 3.13E-08 |
| *MSTRG.14821.1* | 0 | 4.321875515 | 4.321875515 | 5.08E-06 |
| *MSTRG.14654.3* | 0.358079304 | 4.514449814 | 4.15637051 | 0.044889169 |
| *MSTRG.14645.1* | 0.375991228 | 4.52714931 | 4.151158082 | 2.41E-08 |
| *ENST00000417884* | 0.588448586 | 4.704513937 | 4.116065351 | 9.48E-05 |
| *MSTRG.13095.5* | 1.443599697 | 5.534473078 | 4.090873381 | 6.63E-05 |
| *MSTRG.21549.2* | 2.292124404 | 6.362519157 | 4.070394754 | 1.48E-08 |
| *MSTRG.14655.3* | 2.966288702 | 7.027367018 | 4.061078316 | 4.44E-08 |
| *MSTRG.21549.1* | 0.561168075 | 4.620042607 | 4.058874531 | 0.000230074 |

| **Table S4.** Top 20 ceRNA network constructed in 10 stage I LUAD patients | | | | | | | | | | |
| --- | --- | --- | --- | --- | --- | --- | --- | --- | --- | --- |
| **ceRNA_id** | **mRNA_id** | **LncRNA_ID** | **Symbol** | **Num_of_miRNA**  **in_ceRNA** | | **Num_of_miRNA**  **in_mRNA** | **Num_of_miRNA**  **in_ceRNA &mRNA** | **Shared_miRNAs** | **PPC** | **PPC_*P* value** |
| MSTRG.7694.1 | ENST00000331343 | ENST00000554732 | *POFUT2* | | 29 | 259 | 3 | hsa-miR-1226-3p, hsa-miR-210-5p, | 0.98983 | 5.55E-17 |
|  |  |  |  |  |  |  |  | hsa-miR-331-3p |  |  |
| MSTRG.7693.2 | ENST00000398174 | ENST00000554732 | *HM13* | | 18 | 207 | 2 | hsa-miR-141-5p, hsa-miR-216a-3p | 0.988227 | 2.22E-16 |
| MSTRG.7694.1 | ENST00000398174 | ENST00000435813 | *HM13* | | 29 | 207 | 2 | hsa-miR-1226-3p, hsa-miR-216a-3p | 0.985769 | 1.11E-15 |
| MSTRG.10260.1 | ENST00000331343 | ENST00000593604 | *POFUT2* | | 61 | 259 | 6 | hsa-miR-1226-3p, hsa-miR-210-5p, | 0.985469 | 1.33E-15 |
|  |  |  |  |  |  |  |  | hsa-miR-331-3p, hsa-miR-4758-3p, |  |  |
|  |  |  |  |  |  |  |  | hsa-miR-6815-5p, hsa-miR-92b-5p |  |  |
| MSTRG.7694.1 | ENST00000621161 | ENST00000554732 | *RAB11FIP4* | | 29 | 385 | 6 | hsa-miR-1226-3p, hsa-miR-216a-3p, | 0.983657 | 3.66E-15 |
|  |  |  |  |  |  |  |  | hsa-miR-331-3p, hsa-miR-486-3p, |  |  |
|  |  |  |  |  |  |  |  | hsa-miR-508-5p, hsa-miR-5698 |  |  |
| MSTRG.7693.2 | ENST00000621161 | ENST00000554732 | *RAB11FIP4* | | 18 | 385 | 3 | hsa-miR-216a-3p, hsa-miR-508-5p, | 0.982108 | 8.38E-15 |
|  |  |  |  |  |  |  |  | hsa-miR-5698 |  |  |
| MSTRG.10260.1 | ENST00000398174 | ENST00000593604 | *HM13* | | 61 | 207 | 5 | hsa-miR-1226-3p, hsa-miR-216a-3p, | 0.979249 | 3.15E-14 |
|  |  |  |  |  |  |  |  | hsa-miR-4446-3p, hsa-miR-4532, h |  |  |
|  |  |  |  |  |  |  |  | sa-miR-6815-5p |  |  |
| MSTRG.10261.1 | ENST00000331343 | ENST00000554732 | *POFUT2* | | 18 | 259 | 3 | hsa-miR-210-5p, hsa-miR-331-3p, | 0.976773 | 8.59E-14 |
|  |  |  |  |  |  |  |  | hsa-miR-449b-3p |  |  |
| MSTRG.10260.1 | ENST00000621161 | ENST00000554732 | *RAB11FIP4* | | 61 | 385 | 9 | hsa-miR-1226-3p, hsa-miR-216a-3p, | 0.975704 | 1.28E-13 |
|  |  |  |  |  |  |  |  | hsa-miR-331-3p, hsa-miR-4281, |  |  |
|  |  |  |  |  |  |  |  | hsa-miR-4446-3p, hsa-miR-4758-3p, |  |  |
|  |  |  |  |  |  |  |  | hsa-miR-486-3p, hsa-miR-508-5p, |  |  |
|  |  |  |  |  |  |  |  | hsa-miR-5698 |  |  |
| MSTRG.11201.2 | ENST00000379090 | ENST00000554732 | *ZNF774* | | 6 | 238 | 1 | hsa-miR-508-5p | 0.97121 | 5.81E-13 |
| MSTRG.5402.1 | ENST00000339824 | ENST00000554732 | *KSR2* | | 17 | 662 | 4 | hsa-miR-216a-3p, hsa-miR-4446-3p, | 0.970925 | 6.35E-13 |
|  |  |  |  |  |  |  |  | hsa-miR-486-5p, hsa-miR-508-5p |  |  |
| MSTRG.8730.1 | ENST00000621161 | ENST00000554732 | *RAB11FIP4* | | 23 | 385 | 1 | hsa-miR-508-5p | 0.969991 | 8.40E-13 |
| MSTRG.8730.1 | ENST00000331343 | ENST00000593604 | *POFUT2* | | 23 | 141 | 2 | hsa-miR-210-5p, hsa-miR-6815-5p | 0.969473 | 9.79E-13 |
| MSTRG.8730.1 | ENST00000398174 | ENST00000593604 | *HM13* | | 23 | 207 | 2 | hsa-miR-4532, hsa-miR-6815-5p | 0.967387 | 1.76E-12 |
| MSTRG.26440.2 | ENST00000331343 | ENST00000593604 | *POFUT2* | | 99 | 259 | 6 | hsa-miR-1226-3p, hsa-miR-210-5p, | 0.967336 | 1.79E-12 |
|  |  |  |  |  |  |  |  | hsa-miR-331-3p, hsa-miR-449b-3p, |  |  |
|  |  |  |  |  |  |  |  | hsa-miR-4758-3p, hsa-miR-6815-5p |  |  |
| MSTRG.10260.1 | ENST00000269391 | ENST00000554732 | *RNF157* | | 61 | 236 | 7 | hsa-miR-296-3p, hsa-miR-331-3p, | 0.965277 | 3.07E-12 |
|  |  |  |  |  |  |  |  | hsa-miR-4284, |  |  |
|  |  |  |  |  |  |  |  | hsa-miR-449a, hsa-miR-4634, |  |  |
|  |  |  |  |  |  |  |  | hsa-miR-4758-3p, |  |  |
|  |  |  |  |  |  |  |  | hsa-miR-508-5p |  |  |
| MSTRG.11201.2 | ENST00000621161 | ENST00000554732 | *RAB11FIP4* | | 6 | 385 | 1 | hsa-miR-508-5p | 0.965149 | 3.17E-12 |
| MSTRG.10260.1 | ENST00000339824 | ENST00000554732 | *KSR2* | | 61 | 662 | 11 | hsa-miR-210-5p, hsa-miR-216a-3p, | 0.964964 | 3.33E-12 |
|  |  |  |  |  |  |  |  | hsa-miR-331-3p,hsa-miR-345-5p, |  |  |
|  |  |  |  |  |  |  |  | hsa-miR-4443, hsa-miR-4446-3p, |  |  |
|  |  |  |  |  |  |  |  | hsa-miR-4758-3p, hsa-miR-508-5p, |  |  |
|  |  |  |  |  |  |  |  | hsa-miR-5698,hsa-miR-654-5p, |  |  |
|  |  |  |  |  |  |  |  | hsa-miR-6882-5p |  |  |
| MSTRG.10261.1 | ENST00000339824 | ENST00000554732 | *KSR2* | | 18 | 662 | 3 | hsa-miR-210-5p, hsa-miR-331-3p, | 0.964507 | 3.73E-12 |
|  |  |  |  |  |  |  |  | hsa-miR-449b-3p |  |  |
| MSTRG.10261.1 | ENST00000621161 | ENST00000554732 | *RAB11FIP4* | | 18 | 385 | 1 | hsa-miR-331-3p | 0.963417 | 4.88E-12 |

| **Table S5.** Hub lncRNAs predicted by WGCNA using TCGA dataset | |
| --- | --- |
| **lncRNAs** | **Modules in WGCNA** |
| *LINC00639* | MEgrey60 |
| *LA16c-329F2.1* | MEgrey60 |
| *LINC01117* | MEgrey60 |
| *RP11-595B24.2* | MEred |
| *C2orf48* | MEred |
| *CTD-2576D5.4* | MEred |
| *RP11-339B21.10* | MEred |
| *RP11-363N22.2* | MEred |
| *RP11-486M23.1* | MEroyalblue |
| *RP4-676L2.1* | MEroyalblue |
| *RP11-390B4.3* | MEroyalblue |
| *RP11-548P2.2* | MElightgreen |
| *CTD-2349P21.9* | MElightgreen |
| *LINC01468* | MEdarkgreen |
| *LINC00592* | MEdarkgreen |
| *RP11-215P8.4* | MEdarkgreen |
| *HID1-AS1* | BLACK |
| *RP11-251M1.1* | BLACK |
| *FENDRR* | BLACK |
| *RP11-371A19.2* | BLACK |
| *RP1-18D14.7* | BLACK |
| *RP11-286H15.1* | BLACK |
| *CASC2* | YELLOW |
| *RP11-193M21.1* | YELLOW |
| *AC013264.2* | YELLOW |
| *CTC-441N14.1* | YELLOW |
| *RP11-295M3.4* | YELLOW |
| *CTC-441N14.2* | YELLOW |
| *LINC01552* | YELLOW |
| *RP11-268F1.3* | YELLOW |
| *CTD-2531D15.4* | YELLOW |
| *RP11-23J9.4* | YELLOW |
| *VWA8-AS1* | YELLOW |
| *SRGAP3-AS2* | YELLOW |
| *RP11-424M24.5* | YELLOW |
| *C11orf97* | YELLOW |
| *RP11-356K23.1* | YELLOW |
| *RP5-978I12.1* | YELLOW |
| *AC008278.2* | YELLOW |
| *RP4-547N15.3* | YELLOW |
| *RP4-539M6.14* | YELLOW |
| *RP11-196E1.3* | YELLOW |
| *CTB-51J22.1* | YELLOW |
| *RP4-666F24.3* | YELLOW |
| *RP11-122M14.1* | YELLOW |
| *CTD-2562J15.6* | YELLOW |
| *RP11-30O15.1* | YELLOW |
| *HHATL-AS1* | YELLOW |
| *RP11-275I14.4* | YELLOW |
| *ARMC2-AS1* | YELLOW |
| *RP11-503N18.4* | YELLOW |
| *CTD-2544H17.1* | YELLOW |
| *RP11-60L3.1* | YELLOW |
| *RP11-428C19.5* | YELLOW |
| *RP11-57H12.3* | YELLOW |
| *RP11-161H23.9* | YELLOW |
| *LINC01513* | YELLOW |
| *RP11-60L3.6* | YELLOW |
| *AC093159.1* | YELLOW |
| *RP11-356K23.2* | YELLOW |
| *CTD-2126E3.3* | YELLOW |
| *AQP4-AS1* | YELLOW |
| *UMODL1-AS1* | YELLOW |
| *RP11-128M1.1* | YELLOW |
| *AC104809.2* | YELLOW |
| *RP11-428C19.4* | YELLOW |
| *FLJ26850* | YELLOW |
| *LINC01267* | YELLOW |
| *LINC01571* | YELLOW |
| *SMC2-AS1* | YELLOW |
| *CTD-2105E13.15* | YELLOW |
| *RP11-368I7.4* | YELLOW |
| *RP11-647F2.2* | YELLOW |
| *AC011899.10* | YELLOW |
| *AC195454.1* | YELLOW |
| *RP11-568A7.3* | YELLOW |
| *TTLL10-AS1* | YELLOW |
| *RP11-368I7.6* | YELLOW |
| *RP11-394B2.6* | YELLOW |
| *UCKL1-AS1* | YELLOW |
| *RP11-473M20.11* | YELLOW |
