# Supplementary figures and images for: An integrated analysis of the competing endogenous RNA network associated of prognosis of stage I lung adenocarcinoma
Source: BMC Cancer. 2022 Feb 19;22:188. doi: 10.1186/s12885-022-09290-0 (PMC8857797; doi:10.1186/s12885-022-09290-0)

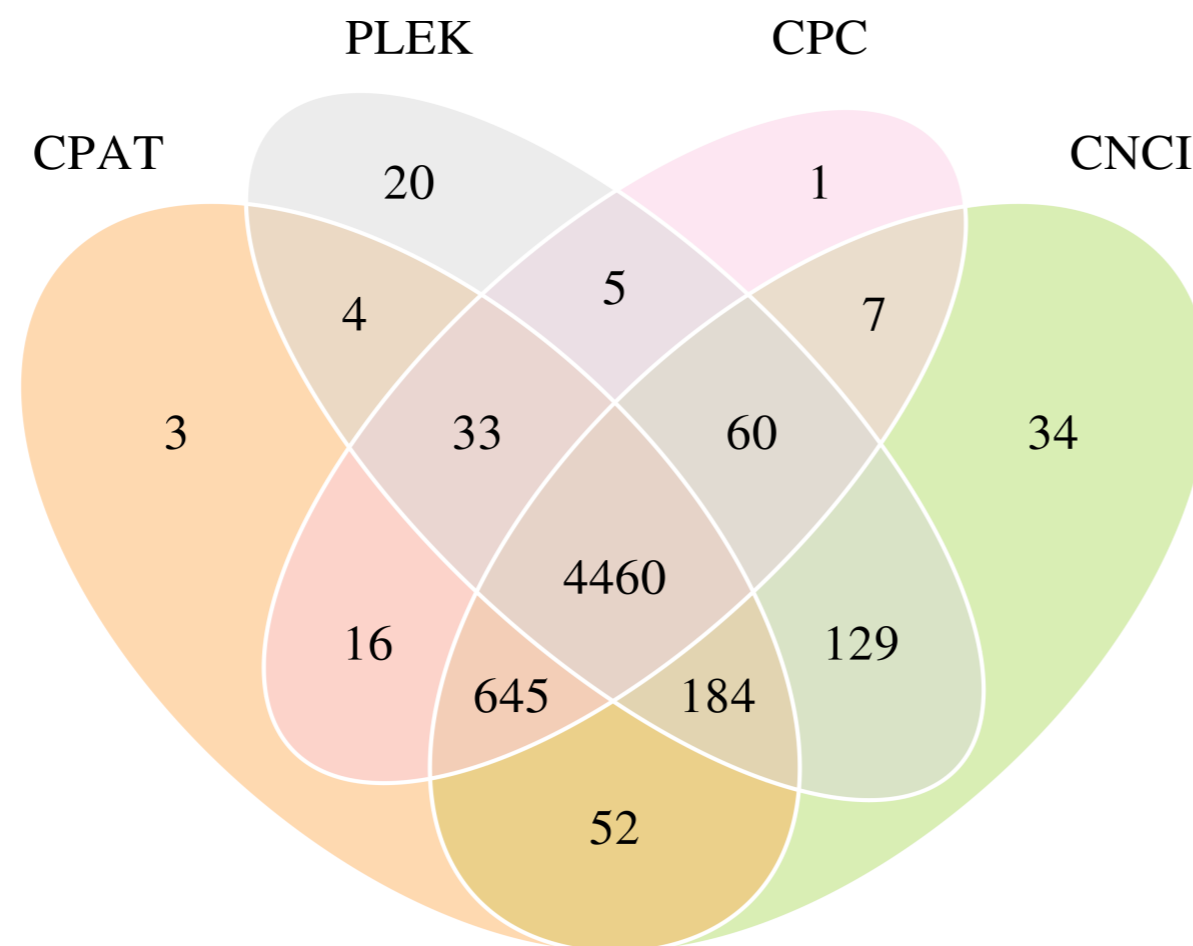

Supplement: Supplementary file 2 — Additional file 2. [file 12885_2022_9290_MOESM2_ESM.pdf]

A

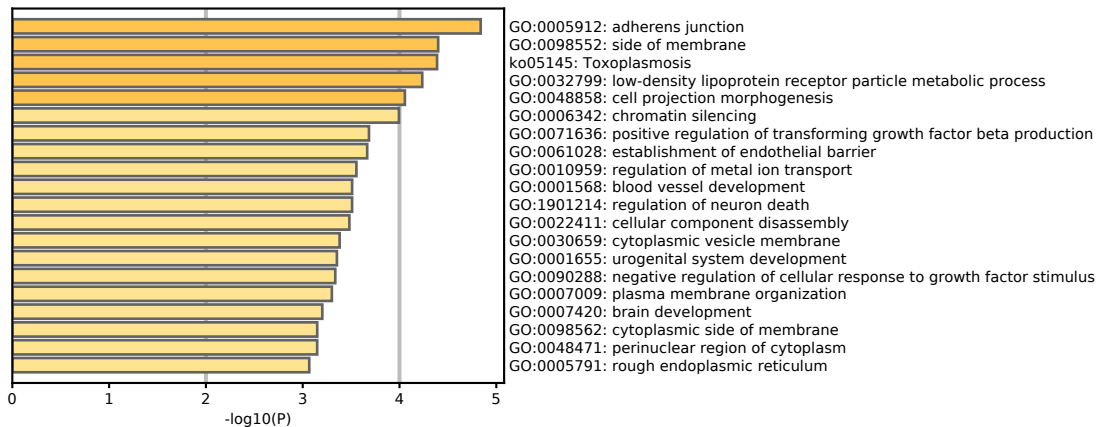

B

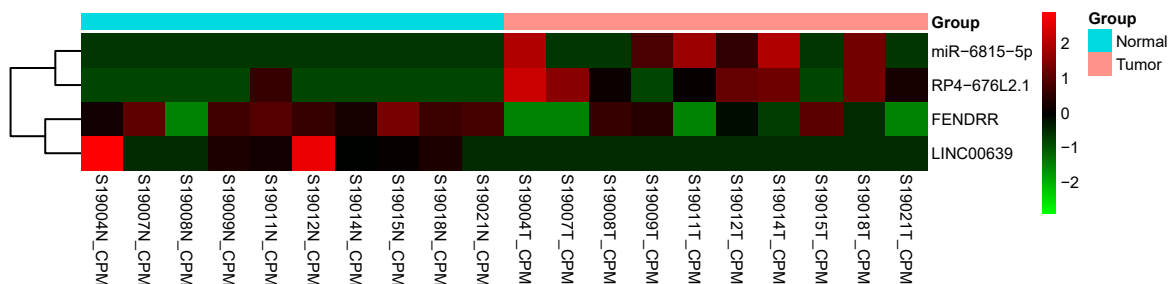

C

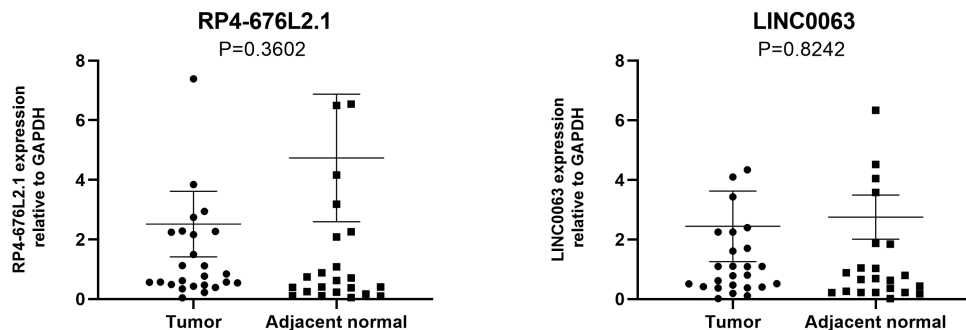

D

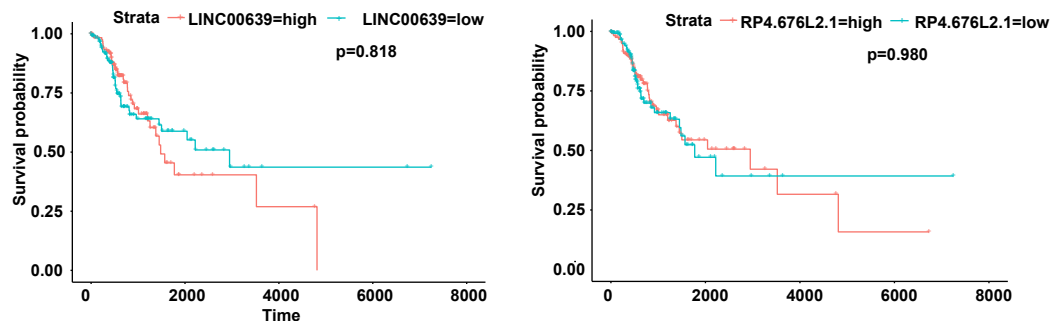

Supplement: Supplementary file 3 — Additional file 3. [file 12885_2022_9290_MOESM3_ESM.pdf]

P=0.0044

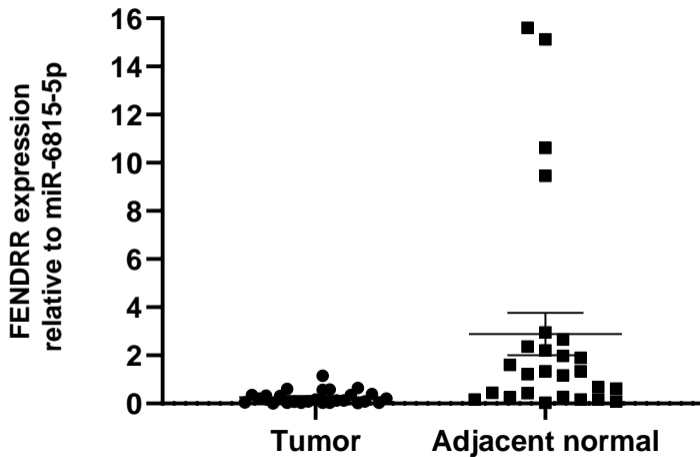

Supplement: Supplementary file 4 — Additional file 4. [file 12885_2022_9290_MOESM4_ESM.pdf]
